# Effects of human activities on *Sericinus montela* and its host plant *Aristolochia contorta*

**DOI:** 10.1038/s41598-023-35607-5

**Published:** 2023-05-22

**Authors:** Si-Hyun Park, Jae Hyun Kim, Jae Geun Kim

**Affiliations:** 1 Department of Biology Education, Seoul National University, Seoul, 08826 Republic of Korea; 2 Warnell School of Forestry & Natural Resources, University of Georgia, Athens, GA 30602 USA; 3 Center for Education Research, Seoul National University, Seoul, 08826 Republic of Korea

**Keywords:** Ecology, Ecology, Environmental sciences

## Abstract

*Sericinus montela,* a globally threatened butterfly species, feeds exclusively on *Aristolochia contorta* (Northern pipevine)*.* Field surveys and glasshouse experiments were conducted to obtain a better understanding of the relationship between the two species. Interviews with the persons concerned with *A. contorta* were conducted to collect information about the site management measures. We found that management practices to control invasive species and manage the riverine areas might reduce the coverage of *A. contorta* and the number of eggs and larvae of *S. montela*. Our results indicated that the degraded quality of *A. contorta* may result in a decrease in *S. montela* populations by diminishing their food source and spawning sites. This study implies that ecological management in the riverine area should be set up to protect rare species and biodiversity.

## Introduction

Butterflies significantly contribute to ecosystem biodiversity^1–3^, providing various ecological benefits such as pollination and natural pest control. Furthermore, butterflies can serve as indicator species to assess and interpret the status of an ecosystem^4–7^. However, butterflies are currently highly threatened due to habitat degradation and loss as a result of human activity^8–10^. Humans are encroaching on and destroying wildlife habitats for their own needs at an alarming rate^11–14^. Weeds and pests are some of the main targets for elimination in a human-centered ecosystem^15^. In agroforestry industries, pesticides have been extensively used to control pests on crops, resulting in ecological collapse^16–18^. Butterflies are sensitive to human activities^19, 20^, as they lay their eggs on their host plants or nearby in areas populated by people. The presence, abundance, and vitality of host plants can influence the survival and diversity of butterfly populations^21^. In the larval stage, butterflies are in danger if host plants are disturbed by human activities such as applying pesticides, mowing, and weeding, or grazing and trampling by livestock^22^. Understanding the quality of host plants is an important step in estimating the abundance and status of butterflies and establishing management practices for butterfly conservation.

The dragon swallowtail butterfly (*Sericinus montela*) is designated as a vulnerable species on a regional scale in South Korea according to IUCN criteria^23^. *S. montela* is known for the beauty of its unique wings, which have a pair of slender tails elongated from each hindwing^24, 25^. These butterflies have a life cycle of 36–54 days including the egg stage (5–7 days), larval stage (15–25 days), pupal stage (10–12 days), and adult stage (7–12 days)^25^. Its larvae are monophagous, feeding exclusively on Northern pipevine (*Aristolochia contorta*), a perennial herbaceous vine species. This plant is a stem-twiner, but is ground-rooted, non-parasitic, and non-epiphytic^26^. Usually, *A. contorta* grows on the edges of forests, rivers, and agricultural fields in East Asia (Korea, Japan, China, and Russia)^27–29^. It has low sexual reproductivity and forms small populations^30^. One of the reasons why *A. contorta* populations are in decline may be the transition from traditional agricultural practices to modern methods, including the use of pesticides, herbicides, and weeding^27–32^. In addition, river improvement works, human-made changes to improve navigation, drainage, irrigation, or flood control, may have also contributed to the decline of its population^33^. These activities could have disrupted the natural habitat of *A. contorta*, making it difficult for this species to survive and reproduce in time. The decline of the *A. contorta* population may result in a decrease of the *S. montela* population, since *S. montela* relies on the plant as the only food source for its larvae.

There are studies on the ecological importance of *A. contorta* and *S. montela* focusing on functional aspects of the plant (i.e., its secondary metabolite)^34–37^, as well as the mitochondrial genome, development, and metapopulation dynamics of the butterfly^38–40^. Recently, the optimal habitat of *A. contorta*^29^ and the interactive effects of CO_2_ on *A. contorta* for *S. montela*^41, 42^ were reported. Currently, none of the studies have examined the cumulative effect of anthropogenic disturbances on *A. contorta* and *S. montela* beyond one-year life cycle. To address this gap, we investigated the anthropogenic factors that can damage the habitat of *A. contorta* using a four-year interval field survey. It involved interviews with stakeholders such as government officers, laborers, and farmers who were directly involved in land management practices that may affect the survival of *S. montela* populations. Furthermore, we performed a glasshouse experiment to examine the effects of the disturbed *A. contorta* on *S. montela*. We hypothesized that (1) mowing and pesticides will negatively impact the growth and reproduction of *A. contorta*, and (2) the population of *S. montela* will be negatively affected by the decline in growth and reproduction of *A. contorta* resulting from human activities. By identifying the factors contributing to the population decline of *S. montela*, this study can suggest management practices that promote the survival and conservation of the species. The findings of this study could be used to develop management strategies that reduce the negative impact of human activities on the habitat of *A. contorta* and the consequent survival of *S. montela*.

## Results

### Human activity and *A. contorta*

We interviewed stakeholders and summarized the mowing and weeding information into six categories (Table 1). Toward the management of various sites including measures such as removing alien species and preserving ecosystem diversity, information was obtained through a series of interviews. PC was managed by Cheongju City and herbaceous plants were removed around the river three times a year by subcontractors. At MA, all plants except the common hibiscus (*Hibiscus syriacus*), a beloved flower in Korea, and some trees, such as the false acacia (*Robinia pseudoacaci*)*,* and the tree of heaven (*Ailanthus altissima*) were removed, while at JW, the *A. contorta* habitat was managed under financial support from Pyeongtaek City. *A. contorta* was considered for removal at GC, where the plant community was separated by a low fence to protect Solomon’s seal (*Polygonatum stenophyllum*). Selective weeding was performed to remove only invasive plants such as Japanese hop (*Humulus japonicus*) and bur cucumber (*Sicyos angulatus*) at YU, but pesticide use was reported by farmers at JP.

**Table 1:** Information on herbaceous plant management at study sites obtained from interviews.

Site	Frequency	Actors	Method	Scope	Locations	Motivation
PC	Three times (Jun., Aug., Sep.)/year	Officials of the River Disaster Prevention Division (Cheongju City)	Using hands, sickles	Herbaceous plants	River bank, near sports facilities and bike lanes, etc	River maintenance
MA	Once (July or August)	Officials of District Office (Anyang City)	Using hands, mowing machine, sickles	Herbaceous plants	River bank	River maintenance, For *hibiscus syriacus*
JW	Frequently (once every 2 weeks from Jun. to Oct.)	Manager of Ecological education research center (Gyeonggi Province)	Using sickles	Invasive alien plants	River bank, *A. Contorta* habitat	Conservation for *S. Montela*
GC	Three times (Jun., Aug., Sep.)/year	Laborers of River Maintenance Department (Yeoju City)	Using mowing machine	Herbaceous plants Invasive alien trees	Near sports facilities and bike lanes etc	River maintenance
YU	Frequently (once a month from Jun. to Oct.)	Laborers of the Han river business division (Seoul Metropolitan City)	Using hands, sickles	Invasive alien plants	River bank, near sports facilities and bike lanes, etc	River maintenance
JP	Frequently (once a month from Jun. to Oct.)	Farmers	Using a mowing machine, herbicide	Herbaceous plants	Near roads	Road maintenance

According to interviews conducted to investigate the anthropogenic activities affecting *A. contorta*, invasive alien plants were removed through government-led conservation programs at sites PC, MA, JW, GC, and YU; however, the removal involved not only invasive exotic plants but also all other native plants and *A. contorta*. At site JP, private mowing machines and herbicides were used for road maintenance.

**Figure 1:**
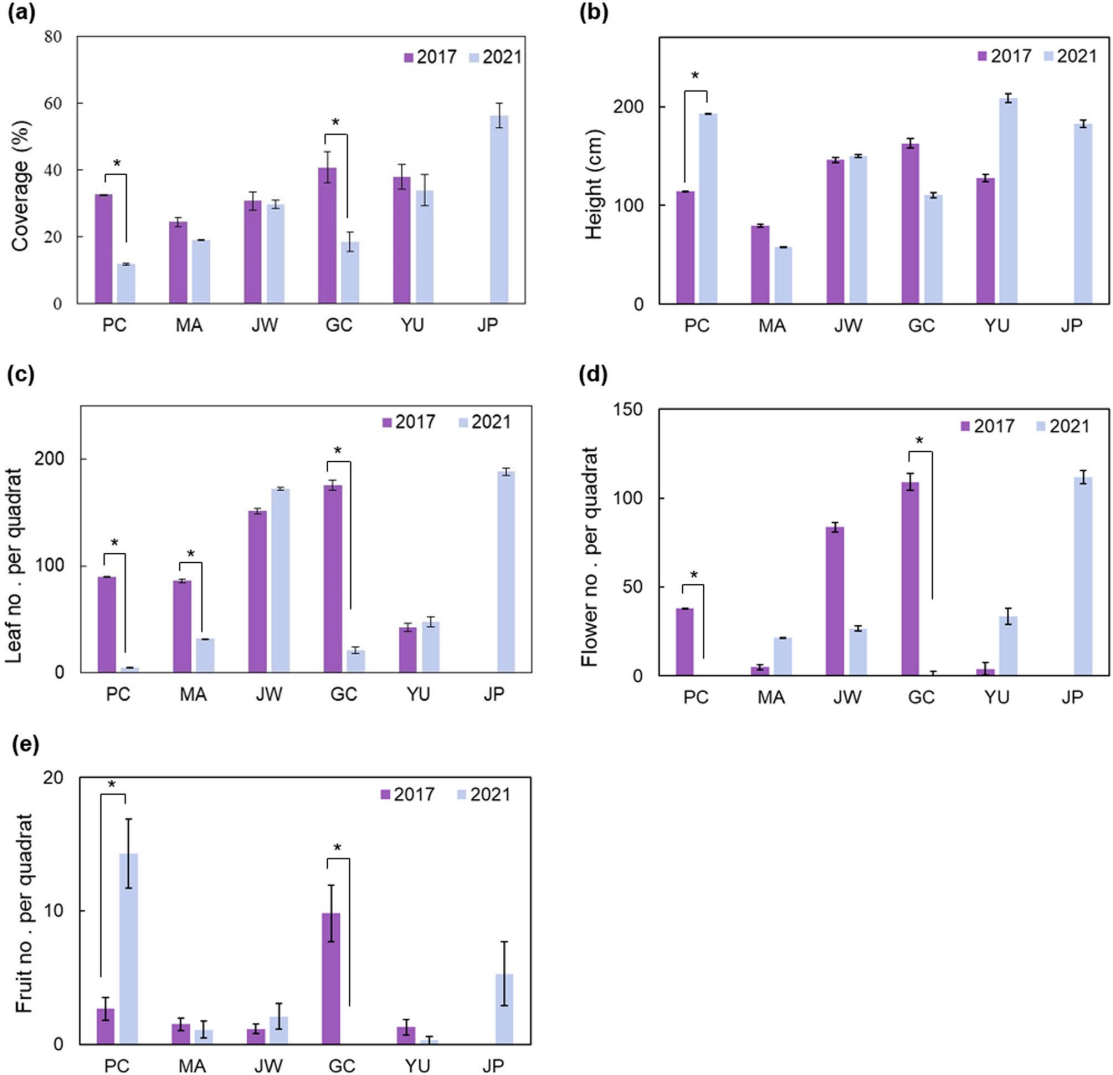
Growth characteristics and reproductive traits of *A. contorta* at study sites in 2017 and 2021 (JP only has data for 2021). (**a**) Coverage, (**b**) Height, (**c**) Leaf number per quadrat, (**d**) Flower number per quadrat, (**e**) Fruit number per quadrat. Bars indicate standard error. *p < 0.05, **p < 0.01, ***p < 0.001.

The coverage of *A. contorta* in 2021 was reduced significantly compared to that observed in 2017 at site PC and GC. In 2021, the coverage were 11.86 ± 0.30% at site PC, 18.50 ± 2.80% at site GC and 32.70 ± 7.67% at site PC, 40.80 ± 8.35% at site GC in 2017 (Fig. 1a). The height of *A. Contorta* increased significantly only at site PC (Fig. 1b). The height of *A. contorta* was uniform at approximately 150 cm at site JW in 2017 and 2021 because they were winding around artificial structures, with a height of 150 cm. When considering *A. contorta* at site MA, they were winding around shrubs (79.50 ± 12.17 cm, *H. syriacus*) in 2017, but only a few vines were left due to mowing (58.10 ± 11.56 cm) in 2021. The number of leaves per quadrat decreased significantly at sites PC, MA, and GC, but it slightly increased at site JW with an artificial planting area for *A. contorta* and at site YU, where only invasive species were cut. For instance, at site PC, the number of leaves per quadrat was 5.1, the lowest in 2021, which showed a steep decline from 90 in 2017, while the study site with the most leaves in 2021 was JP (188.3 ± 28.7). A positive correlation was found between the coverage and the number of leaves per quadrat (r = 0.678, p < 0.01).

In 2021, *A. contorta* displayed higher coverage, height (with the exception of at site JW which had artificial supports), and number of leaves and flowers in well-maintained areas and lower in areas that had intensive mowing or weeding. Coverage was the highest at site JP (56.43 ± 3.64%), which was newly investigated in 2021, followed by at site YU (34.00 ± 4.66%), JW (29.80 ± 1.36%), MA (19.10 ± 3.45%), GC (18.50 ± 2.80%), and PC (56.43 ± 3.64%) (Fig. 1a). In 2021, the highest *A. contorta* was observed at site YU (208.5 ± 32.54 cm), followed by at site PC (192.86 ± 30.84 cm) and then at site JP (182.86 ± 22.44 cm) which were winding around *R. pseudoacacia* and *Morus alba*, respectively (Fig. 1b). However, there were many withered *A. contorta* leaves at site PC, so their coverage was low. The number of leaves was the highest at site JP (188.29 ± 18.68), followed by at site JW (172.50 ± 16.09%), YU (47.03 ± 7.76%), MA (32.00 ± 2.84%), GC (21.20 ± 4.35%), and PC (5.00 ± 0.82%) (Fig. 1a).

The values of the reproductive traits of *A. contorta* were significantly different depending on the years and sites (Fig. 1). At sites PC and GC, there was a 100% decrease in the number of flowers (2017 PC, 38.0 ± 11.0; 2021 PC, 0; 2017 GC, 109.0 ± 27.2; 2021 GC, 0). The number of fruits in 2021 significantly decreased at site GC compared to 2017. The number of flowers per quadrat was the highest at site JP (111.86 ± 28.95) and the lowest at sites PC and GC in 2021 (PC, 0; MA, 21.6 ± 7.5; JW, 26.6 ± 9.3; GC, 0; YU, 33.5 ± 14.2; JP, 111.9 ± 28.9; Fig. 1d). A positive correlation was found between the number of flowers per quadrat and the height (r = 0.340, p < 0.05). The number of fruits per quadrat was the highest at site PC and the lowest at site JW in 2021 (PC, 14.3 ± 3.5; JP, 5.3 ± 2.4; GC, 0; YU, 0.3 ± 0.1; JW, 2.1 ± 0.9) (Fig. 1e).

**Figure 2:**
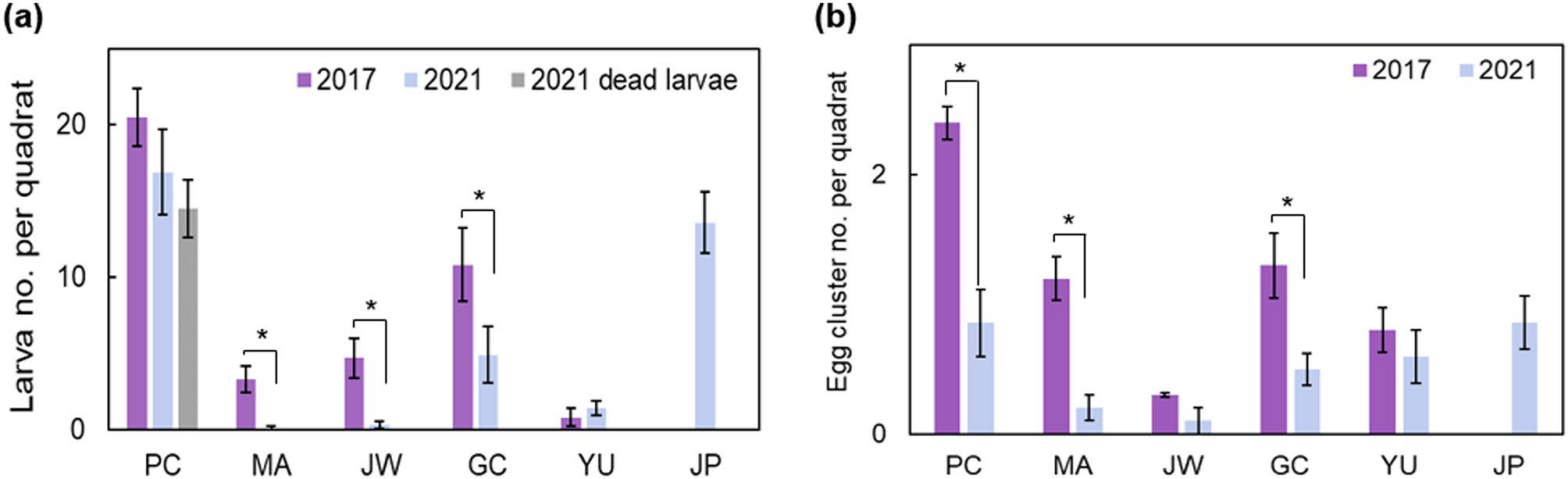
Average number of larvae (**a**) and egg clusters (**b**) of *S. montela* per quadrat at study sites in 2017 and 2021 (JP data was collected in 2021 only). Bars indicate standard error. *p < 0.05, **p < 0.01, ***p < 0.001.

In 2021, the number of larvae decreased at all sites other than YU compared to 2017. In particular, the number of egg clusters was reduced compared to that of 2017 (Fig. 2). At site PC, *S. montela* larvae were distributed in large numbers at a density of 16.9 individuals/m^2^ in 2021 (Fig. 2a). Larvae ate fresh leaves or buds at most sites except for at site PC in 2021, where most leaves had turned brown in August, therefore the larvae gnawed at the surface of fruits. Dead larvae were found dried on the leaves of the host plants (6.6 ± 0.4 individuals/m^2^, Fig. 2a). Due to the lack of leaves, sufficient shade was not provided, therefore the larvae exposed to direct sunlight were likely to die. Also, people, bicycles, and cars killed larvae that reached ground level (8.3 ± 0.2 individuals/m^2^, Fig. 2a).

### Effects of feeding organ and pesticides on the growth of *S. montela*

Before examining the effect of the feeding organ, the C/N ratio of the feeding organ was as follows. The C/N ratio of fruits used in the experiment was 34.02 ± 2.99 (JP), 32.84 ± 3.12 (PC), and the C/N ratio of leaves was 15.82 ± 0.23 (JP), 16.53 (PC), respectively. Among a total of 120 larvae, 75 died before pupation (62.50% larval mortality), eight did not emerge from pupae (6.67% of the initial number of larvae), while 37 emerged (30.83% of the initial number of larvae).

**Figure 3:**
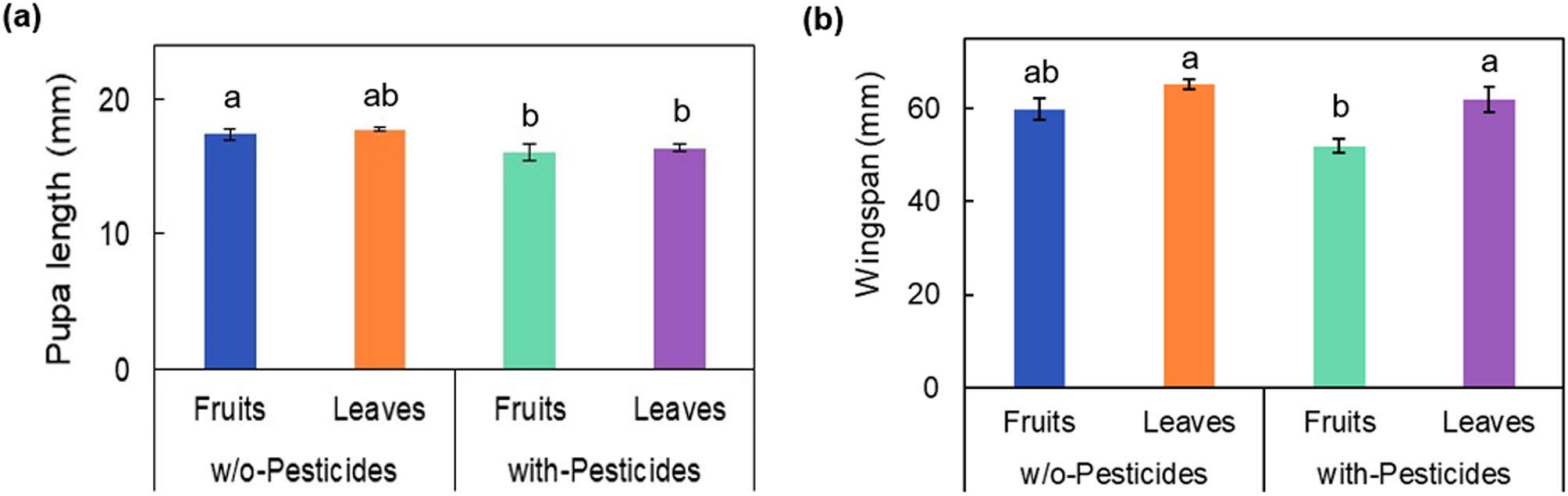
Variations of *S. montela* morphological traits among treatments (w/o-Pesticides: feeding on foods without pesticides, with-Pesticides: feeding on foods with pesticides, Fruits: fruit-eating group, Leaves: leaf-eating group, (**a**) Pupal length, (**b**) Wingspan. (Duncan test, *p* < 0.05). Bars indicate standard error.

**Figure 4:**
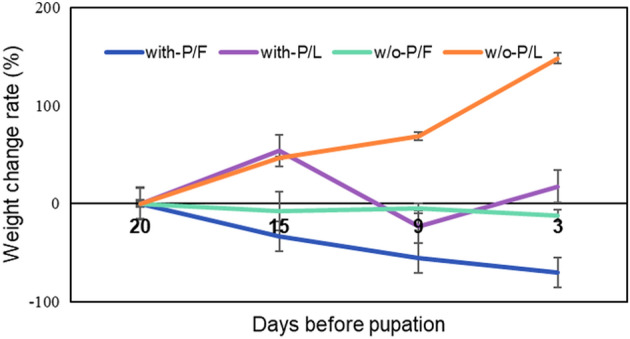
Average growth curves for larvae reared in different treatments (w/o-P/F: feeding on fruits without pesticides, w/o-P/L: feeding on leaves without pesticides, with-P/F: feeding on fruits with pesticides, with-P/L: feeding on leaves with pesticides). Bars indicate standard error.

**Figure 5:**
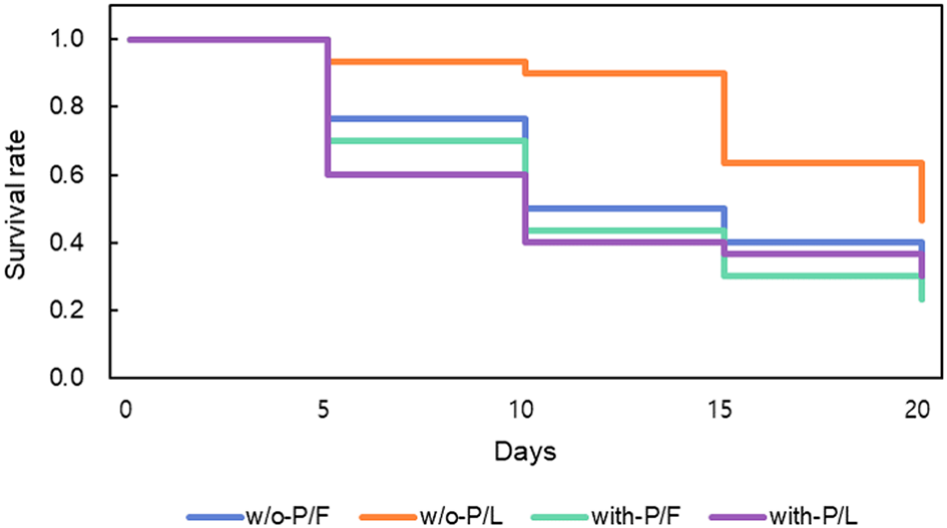
Survival curves of larvae reared in different treatments.

**Figure 6:**
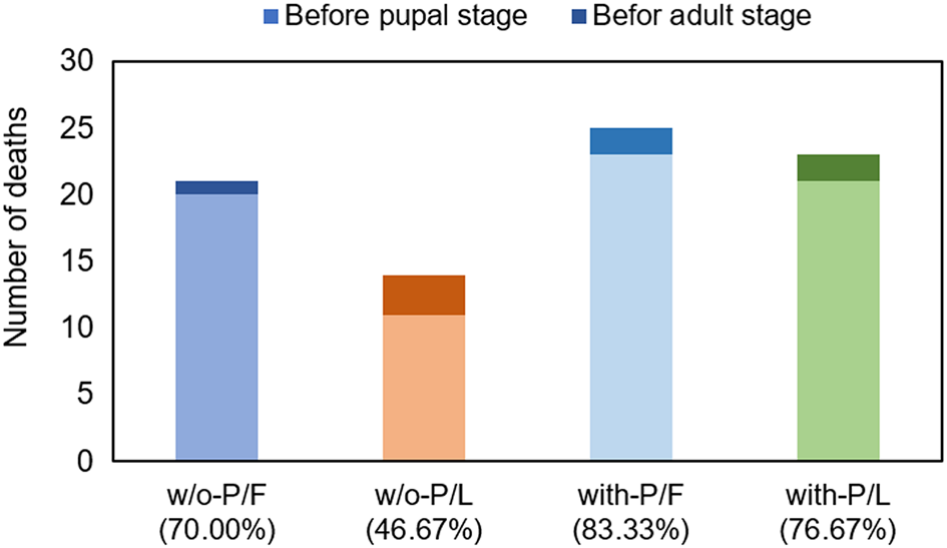
The number of larvae that did not emerge before the pupal stage (light colors) and the number of pupae that did not reach the adult stage (dark colors) under different treatments. The number (%) in parentheses indicates total mortality before the adult stage.

The morphological traits of *S. montela* responded differently according to the food types (leaves or fruits) and the presence or absence of pesticides (Fig. 3). The *S. montela* group fed w/o-Pesticides/Fruits (w/o-P/F) plants had a longer pupal length than those fed with-Pesticides/Fruits (with-P/F). The group fed with-P/L had a longer wingspan than those fed with-Pesticides/Fruits (with-P/F) (Fig. 3). Pupal length and wingspan were the longest in the group of w/o-P/L and were the shortest in the group of w-P/F (Fig. 3). In weight change (%), which is an increase or decrease compared to the original weight of the larva, *S. montela* with-P/F lost more weight than w/o-P/F (Fig. 4). Furthermore, w/o-P/L gained more weight than with-P/L (Fig. 4). In the case of *S. montela* with-P/L, the average weight was temporarily decreased as many large larvae died, but the average weight finally increased (Fig. 4). Furthermore, larvae that fed on fruits showed higher mortality than those fed on leaves (Fruits, 76.67% mortality; Leaves, 61.67% mortality; Kaplan–Meier test Chi-square = 4.596, df = 3, *p-value* = 0.004). In addition, the group fed with-Pesticides food showed higher mortality (80.00%) than those fed on plant matter w/o-Pesticides groups (58.33%; Fig. 5). Larvae fed on fruits with pesticides showed the highest mortality (83.33%). In comparison, those which fed on leaves without pesticides showed the lowest mortality (46.67%; Fig. 6). The comparison of the fractions of dead and emerged individuals from w/o-P/F was 21 vs. 9, while w/o-P/L was 14 vs. 16. In the case of -P/F the comparison between dead and emerged individuals was 25 vs. 5, and in P/L is was 23 vs. 7 with a higher proportion of larvae reaching the adult stage on w/o-P/L than others (Fig. 6). Some larvae did not reach the pupal stage (20 out of 30 w/o-P/F; 11 out of 30 w/o-P/L; 23 out of 30 with-P/F; 21 out of 30 with-P/L). In addition, some pupae did not reach the adult stage (1 out of 10 w/o-P/F; 3 out of 19 w/o-P/L; 2 out of 7 with-P/F; 2 out of 9 with-P/L). There was no mating event observed, but only a few eggs (w/o-P/F, 19, two clusters; w/o-P/L, 35, two clusters; with-P/F, 14, one cluster; with-P/L, 22, one cluster) were laid in the cages.

Pupal length, wingspan, larval weight change, and the number of survival days of *S. montela* depended on the food types and pesticide (Table 2). The pupal length was only affected by pesticides, and the number of survival days was only affected by food types. The wingspan and the larval weight change were affected by the food types and pesticide, respectively. However, there were no interactive effects on pupal length, wingspan, larval weight change, or the number of survival days (Table 2). The differences according to the food types were not apparent with the presence of pesticide and vice versa.

**Table 2:** Two-way ANOVA on traits of *S. montela* (F ratios are shown). Two treatments were food types and the presence of pesticides. df = 1, 43 for pupal length, df = 1, 28 for wingspan, df = 1, 119 for larva weight change, df = 1, 119 for survival days. *p < 0.05, **p < 0.01, ***p < 0.001.

	Food type	Pesticide application	Food type × pesticide application
Pupal length	0.528	10.769**	0.228
Wingspan	12.301**	6.559*	1.024
Larva weight change	126.131***	64.106***	2.108
Days of survival	12.454***	1.199	0.133

Significant values are in bold.

## Discussion

### Response of *A. contorta* to human activities and subsequent effects on the *S. montela* population

*Aristolochia contorta*, the exclusive food source of *S. montela* larvae, mainly occurred along the river bank and in the ecotone between forest and agricultural areas at the surveyed sites. The population size tends to be constantly limited and grows faster as age increases^43^. Furthermore, *A. contorta* grows taller in the presence of trees^29^. The more suitable habitat for *S. montela* is when coverage is higher, stems are more extended, and more leaves are present on *A. contorta*. The coverage and number of *A. contorta* leaves are more significantly correlated with available food resources for butterfly larvae^21^. Consequently, site JP would be the most suitable habitat for *A. contorta* among the study sites (Fig. 1). However, site JP would not be a good habitat for *S. montela* due to pesticide application (Figs. 2, 6).

*Aristolochia contorta* was easily removed during mowing or weeding. In the process of weeding invasive species, *A. contorta* instead of being protected, was also being removed. This occurred through various mechanisms, such as the use of non-selective herbicides, mechanical removal methods, or changes in ecosystem processes resulting from the removal of invasive species. The negative impacts of invasive species management on non-target species can be mitigated through a systematic approach to conservation planning and management that prioritizes biodiversity conservation^44^. This approach involves identifying and prioritizing areas that are critical for biodiversity conservation, including the habitats of vulnerable and threatened species, and incorporating this information into the planning and management of invasive species^44^.

Furthermore, the removal of invasive alien trees such as *R. pseudoacacia* in the same period, resulted in a loss of support structures which is one of the critical factors of *A. controta* growth and reproduction^29^. A management strategy that promotes the restoration of support structures may be necessary. One potential approach could be the planting of native trees or shrubs with similar branching structures as *R. pseudoacacia* in the area that can provide similar support structures^29^. Additionally, artificial support structures such as stakes or trellises could be used to provide support for individual plants until they are able to develop their own support structures^29^. Moreover, the mowing period overlapped with the flowering period of *A. contorta*. After mowing, the roots sprout again, but they may not grow high enough to flower. Because the higher the *A. contorta*, the better the flowers bloom^29^. Consequently, the mowing could affect reproductive traits, inhibiting sexual reproduction by *A. contorta*. However, the rate of asexual reproduction increased due to regular mowing, and new aboveground parts of *A. contorta* regenerated after sustaining damage from mowing. Perennial plants generally tend to reproduce new individuals through asexual reproduction from underground rhizomes or root shoots if the aboveground parts are damaged^45^. If there are human activities during the transitional period before flowering, the reproduction rate of *A. Contorta* could plummet and asexual reproduction could be active^30^. Thus, anthropogenic activities may facilitate asexual reproduction, thereby reducing the genetic diversity of *A. Contorta*. In fact, currently, the genetic diversity of *A. contorta* in South Korea is very low compared to *A. contorta* in Russia, China, and Japan^30, 46^.

In the places where mowing did not occur, other factors might have affected *A. contorta* growth. For instance, high stress resulting from interspecies competition could also bring changes in flowering and fruiting timing in plants^47^. Our results at site GC might indicate that interspecies competition (in this case with an invasive vine plant, *S. angulatus*) can affect the growth of the *A. contorta* population. Interspecies competition may encourage the deterioration of the growth condition of *A. contorta*.

Unexpectedly, the population of *A. contorta* at site PC showed more vigorous growth than at the other study sites: Flowering and fruiting were completed in early August 2021. In Korea, *A. contorta* blooms from July to August, and fruits appear from September to October^48^. Stressed plants eaten by herbivorous insects may engage in rapid flowering and fruiting^49^. High foraging pressure caused by crowding *S. montela* larvae may increase the stress of *A. contorta* individuals. As such, plant stress resulting from insect herbivory could be a factor accelerating the flowering period of plants^50^. Stress may have stimulated the early flowering and fruiting of plant, so the leaves were wilted early in the *A. contorta* population at site PC.

Larvae generally do not leave their host plants before reaching the third instar, except for early-stage larvae swept away by heavy rain^22^. It can be inferred that the larvae at site PC were looking for food due to the lack of fresh leaves by cutting the stem (personal observation of PSH). In this way, the changes in the vegetation of *A. contorta* may affect the population of *S. montela* (Fig. 3). Studies on invertebrates demonstrated a strong correlation between the abundance of phytophagous insects and edible biomass for larval resource^21, 51, 52^. Curtis et al.^21^ found that butterfly abundance was determined by food availability and was mediated by species traits. Especially, monophagous species with narrow diet breadth are far more likely to be resource-limited than polyphagous species^53, 54^. In addition, monophagous species are vulnerable to serving as a buffer for the lack of preferred plants, so the abundance of monophagous *S. montela* could be directly affected by host plant *A. contorta* availability. Management of host plants of target butterfly species can increase population abundance^55^. This, in turn, may allow individual species to attain higher population densities, thereby reducing the risk of extinction and consequently increasing the species abundance of the region^56, 57^.

An interview revealed that most of the growth and reproduction changes in *A. contorta* compared to those observed 4 years ago were due to anthropogenic activities. Because most interviewees were unaware of *A. contorta* and *S. montela,* selective weeding was performed frequently but not discriminately except at sites JW and YU. The selective weeding of invasive plants may positively affect the growth of *A. contorta*. Therefore, it is necessary to notify the weeders of the existence of *A. contorta* and *S. montela* and to tell them not to remove these plants because they should be protected.

### The life history variation of *S. montela* under feeding conditions

In this study we investigated whether the types of plant organs the butterflies feed on and the application of pesticides on the food resources can affect the growth of *S. montela*. Wingspan, weight change, and survival rate of *S. montela* varied depending on the sort of available food (leaves or fruit), which may be related to the C/N ratio. The C/N ratio is considered an essential factor in plant–herbivore interaction. The leaves are usually rich in nitrogen, which is beneficial for the development of herbivorous insects^58–62^ and may consequently be preferred by herbivores. Furthermore, the high-carbon compounds in fruits significantly reduce the larvae's preference^63^. Therefore, when larvae ate fruits with higher carbon, they could not grow properly. When leaves are lacking, *S. montela* may look for some more leaves, requiring more nutrients to reach the adult stage^64^. However, the larvae are likely to be killed while looking for leaves like at site PC (personal observation of PSH).

The pesticides applied to the leaves and fruits used in the experiment were tricyclazole, benfuracarb, and buprofezin. Tricyclazole inhibits pentaketide-derived melanin biosynthesis in fungi and shows AF production–inhibitory activity^65^. Benfuracarb is a broad-spectrum benzofuranyl methylcarbamate insecticide used for crop protection, and it biochemically inhibits acetylcholinesterase activity^66^. Buprofezin, a thiadiazine insecticide, is effective against hemipteran pests and other insects^67^. Buprofezin inhibits chitin synthesis, resulting in molting disruption and abnormal deposition of endocuticles, causing premature death^68, 69^. They are also highly toxic to target and non-target organisms, including insects, mammals, birds, and aquatic organisms^70–74^. The larvae, which had been gaining weight by feeding on with-P/L, suddenly increased their mortality five days after the experiment started (Fig. 5). Because the larvae that fed on a little amount of pesticide-stained leaves and had a smaller weight change survived, the average growth curves for larvae reared in with-P/L suddenly decreased (Fig. 4). Many studies revealed that various pesticides could change the morphological characteristics of insects, such as body length, duration of juvenile development, survival rate, emergence rate, copulation rate, and hatchability^75–77^. Although the molecular mechanism of action of the pesticides could not be confirmed directly in our experiment, it obviously affected the phenotype of *S. montela*.

## Conclusion

This study identified specific threatening factors which were a decrease of feeding organs due to weeding and pesticide spraying to *A. contorta* and *S. montela*. The riverine habitats of *A. contorta* and *S. montela* are likely to get damaged due to inattentive management methods. We suggest systematic conservation planning and management centered on biodiversity conservation to protect and conserve vulnerable species and their habitats even while managing invasive species*.*

## Methods

### Field observations and survey

**Figure 7:**
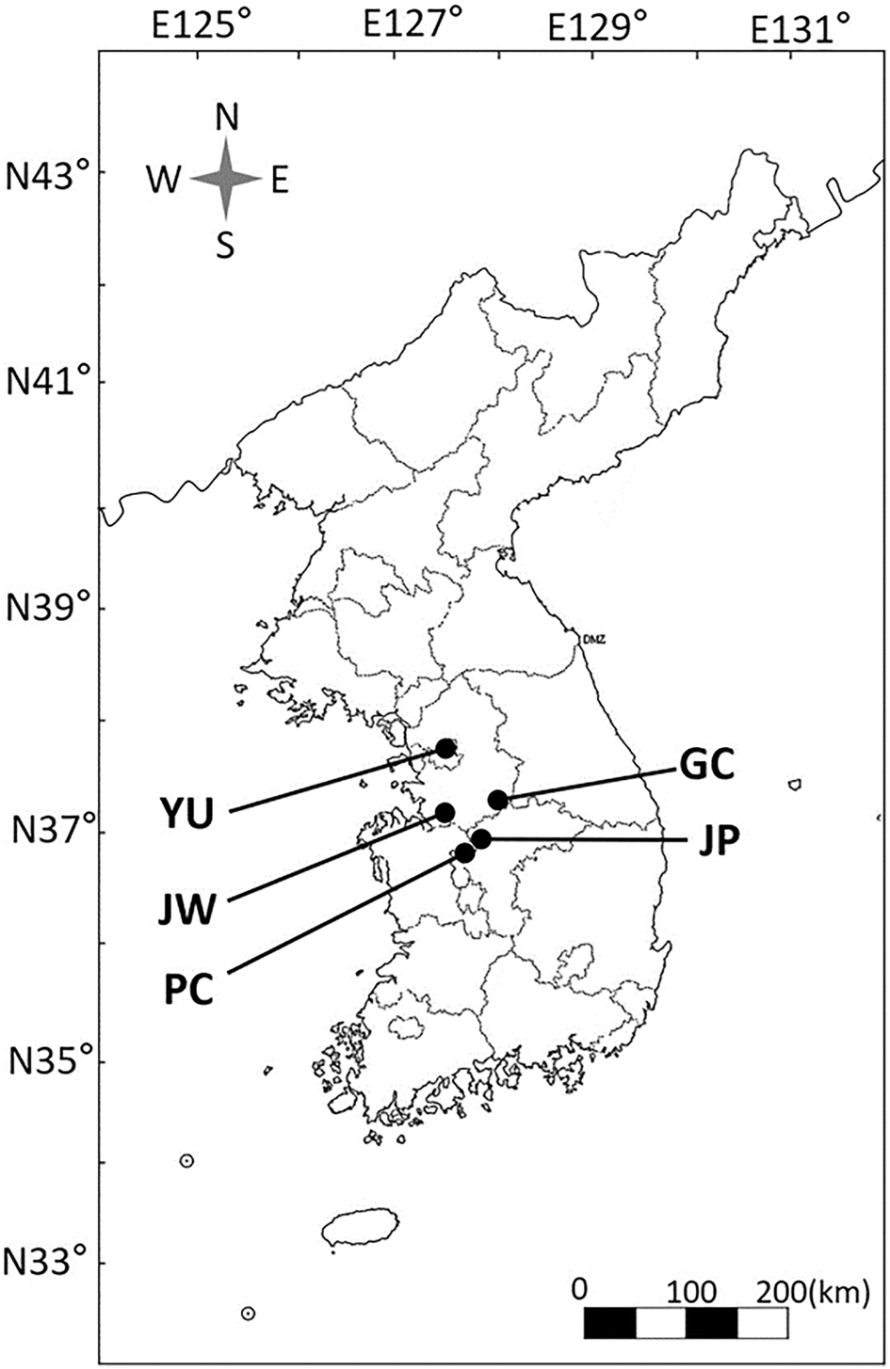
Study sites for field survey in South Korea. *PC* Pyeongchon, *MA* Manan, *JW* Jinwee, *GC* Gangcheon, *YU* Yeouido, *JP* Jeungpyeong.

We investigated *A. contorta* habitats through literature and media reports and selected six sites on roadsides near rivers or rice paddies in South Korea (Fig. 7). Field surveys and observations were conducted from August to November in 2017 and 2021. We added site JP in 2021. Interviews related to habitat management were conducted from August 2021 to February 2022 with 11 stakeholders (1 government officer in each site of PC, MA, JW, GC, YU, 1 research center in JW, 1 subcontractor in GC, 2 laborers in MA, YU, 2 farmers in JP). Environmental properties (presence of accompanying invasive species and coverage of the invasive plant) and plant growth were surveyed with ten quadrats of 1 m × 1 m at each site in the same location in the years 2017, and 2021. The area of the population was different for each site, but each quadrat was at least 5 m apart. The coverage of all plant species was calculated by dividing the area of each species by the area of the quadrat (Coverage (%) = The covered area of each species/The area of the quadrat). Height was measured using the tallest *A. contorta* individual in the quadrat. The number of leaves and flowers of *A. contorta* was counted in each quadrat in August 2017 and 2021, and the number of fruits was counted in each quadrat in November 2017 and 2021 (the same site was investigated on a similar date). The number of eggs, larvae, and dead larvae of *S. montela* present on the stem and leaves of *A. contorta* and on the ground was recorded. Field observations were conducted to record any disturbances on host plants, such as mowing, weeding, trampling, and pesticide application. In association with direct observation, we interviewed laborers, city officials, and local people on the sites to record information on the past and present status of the habitat, threats, and conservation of the study areas.

We identified plants with illustrated plant books of Lee^48^ and Flora of Korea Editorial Committee^78^. S.-H.P took pictures of all the plants for certification according to the national regulation. There is no regulation for the collection of *A. contorta* in Korea and *A. contorta* is regarded as a weed by farmers.

### Experimental setting

Based on field observations, factors that could threaten *A. contorta* were identified. At site JP, the condition of the *A. contorta* habitat was mainly suitable for *S. montela*, but mowing was performed and we captured farmers applying pesticides there. According to the farmers, the pesticides included tricyclazole, benfuracarb, and buprofezin. We collected *A. contorta* leaves and fruits at site JP for the experiment to investigate the effects of mowing and pesticides on the growth of *S. montela*. The C/N ratio is related to food quality, which in turn affects *S. montela* survival rate, and was analyzed with the part of the leaves and fruits using an Elemental Analyzer (Flash EA 1112, Thermo Electron, USA) by NICEM at Seoul National University. Site PC, whose C/N ratio of leaves and fruits was similar to site JP, was selected as the control. In order to exclude the predecessor feeding experience of the larvae in site PC and JP, 120 third-instar larvae, which have a low mortality rate from other causes^22^, were taken from site GC. Experimental settings were established in a test room at Seoul National University from August 2021 to September 2021. Three larvae were put into an insect breeding dish (10 cm diameter and 4 cm depth). Four experimental treatments (30 larvae per treatment) were performed with a factorial array of leaves × fruits of *A. contorta,* and pesticides sprayed × not sprayed. Feeding, molting, and development of larvae were observed in insect breeding dishes at 25 ± 2 °C. Larvae were weighed every 3–5 days, and larval weight change (increase or decrease) compared to their original weight was calculated. The body length of pupae and wingspan were measured using vernier calipers. For eclosions the pupae, glued to sticks were placed in the Butterfly Habitat Terrarium (60 × 60 × 90 cm). The number of deaths from the larval stage and eclosions from the pupae stage were recorded, and mortality rates as well as the number of days *S. montela* survived were calculated. After eclosions, we observed whether they mated and counted the number of eggs. All the experiments were performed in accordance with relevant guidelines (ethical guidelines, animal welfare, data management) and safety regulations.

### Data analysis

To compare the growth and reproductive characteristics of *A. contorta*, and the average number of larvae and egg clusters of *S. montela* in 2017 and 2021, *t*-test at the 5% significance level were conducted after the homogeneity of variance test using SPSS ver.23.0 software (SPSS, Inc., Chicago, IL). Correlation analysis was performed to examine the relationships between variables (the coverage and the number of leaves per quadrat, the number of flowers per quadrat and the height) using SPSS. Pearson's correlation coefficient was used to assess the strength and direction of association between variables. The effects of feeding organs (leaves, fruits) and the effects of pesticides on morphological and reproductive traits of *S. montela* were also analyzed with the same process. The interactions between feeding on leaves × fruits and feeding on foods with pesticides × without pesticides were analyzed with a two-way analysis of variance (ANOVA) after homogeneity of variance was confirmed. Duncan’s test was used for post-hoc analysis.

## Data Availability

The article contains all of the data that were created and used in this investigation, and the corresponding authors can provide you with these data upon request.
